# Does parents’ retirement influence the fertility intentions of subsequent generations among internal migrant populations? Evidence from China

**DOI:** 10.1371/journal.pone.0311028

**Published:** 2024-10-01

**Authors:** Tianxin Cai, Shilong Ma, Renyao Zhong

**Affiliations:** 1 School of Public Management, East China Normal University, Shanghai, China; 2 School of Public Administration and Emergency Management, Jinan University, Guangzhou, China; Flinders University, AUSTRALIA

## Abstract

**Objectives:**

The fertility health of the migrating population has attracted significant attention. This article examines the impact of parental retirement on the fertility intentions of the offspring among the internal migrant population.

**Methods:**

This study utilizes the mandatory retirement age system as an exogenous shock within a fuzzy regression discontinuity (FRD) experimental design to investigate the influence of parents’ retirement on the fertility intentions of their migrant offspring and potential mechanisms.

**Results:**

The research findings indicate that parents’ retirement significantly reduces the fertility intentions of the migrant population by 34.4%. Mechanism analysis attributes this adverse effect to the reduction in intergenerational wealth transfer due to parent’s retirement. As the family’s economic situation worsens, the negative impact of retirement on fertility intentions becomes more pronounced. The childcare support mechanism provided by retired grandparents can partially offset the adverse effects of the income mechanism.

**Conclusions:**

This study provides recommendations for enhancing policies related to delaying retirement age and childcare.

## Introduction

Retirement and childbirth have recently been hot topics in public policy discussions. Increasing research attention is being paid to the intergenerational impact of fertility intentions as birth rates continue to decline [1, 2]. Studies have examined how childcare support, family socialization, and other factors affect fertility intentions across generations [3–6].

Among these studies, a growing body of research has documented that the retirement of the parent generation not only affects their labor supply but also influences the fertility decisions of the subsequent generation [3]. This strand of literature suggests that parents’ retirement may influence the offspring’s fertility decision from three perspectives. First, postponing the statutory retirement age may squeeze out the time for grandparents to provide childcare, thereby inhibiting fertility intentions [6–9]. Second, although both social and family-based elderly care may encroach on fertility resources in an aging society, higher fertility rates may be achieved if delayed retirement leads to greater output and complementary social resources in support of childbirth [10, 11]. Additionally, the impact of delayed retirement on fertility depends on specific circumstances. Studies deduced through a unified growth theory that if parents prioritize the quality of their children, delayed retirement could reduce the number of children in a family [12]. Conversely, delayed retirement might increase a family’s fertility level if parents prioritize quantity over quality. In conclusion, the academic community has reached a general consensus on intergenerational influences on fertility intentions. However, there remains some controversy regarding the impact of retirement on the fertility intentions of the subsequent generation and the underlying mechanisms.

Despite the insightful findings, limited discussions exist on the intergenerational impact of fertility intentions among migrants. China’s internal migrant population, distinct from international migrants, is substantial and possesses unique characteristics. Unlike international migration, which involves crossing national borders, internal migration in China refers to the large-scale movement of people within the country, typically from rural to urban areas or between provinces, often driven by economic opportunities and urbanization processes [13]. This population, while not crossing international borders, faces many challenges similar to international migrants due to China’s *hukou* system. The data from the Seventh National Population Census reveals that in the year 2020, the scale of the internal migrant population was approximately 380 million people. Due to the separation between the place of residence and registered hometown, the internal migrant population may lose certain welfare benefits available in their registered hometowns, particularly in areas such as children’s education, healthcare, and housing. This fact is especially true in China, where the provision of local services is closely tied to hukou policies. Consequently, internal migrants may face higher childbirth costs and elderly care than local residents. Thus, migrants’ fertility intentions may be influenced differently by their parents’ retirement than their local counterparts, which is worthwhile to explore.

Drawing upon the current literature, we argue that their parents’ retirement may influence migrants’ fertility intentions through two main mechanisms: the childcare support mechanism and the income mechanism. On the one hand, migrants’ fertility intentions may be increased due to the increased childcare offered by their retired parents, which we refer to as the "childcare support mechanism." Grandparental care, particularly in China, plays a critical role in the family structure, where grandparents often take on significant responsibilities in raising grandchildren. This practice is deeply rooted in traditional cultural norms and is further necessitated by the high cost and scarcity of formal childcare services. Current literature suggests that childcare provided by grandparents has become one of the significant sources of informal childcare [14, 15], which reduces the costs and risks of childbearing and thus has a positive correlation with fertility intentions of the subsequent generation [16]. This intergenerational support, however, varies significantly across different countries and cultures. For instance, in Western countries, where childcare services are of high quality and affordable and individual independence is important, social expectations regarding grandparenting are low, whereas in East Asian contexts like China, older adults are perceived as having an obligation to provide care to their grandchildren [17].

On the other hand, migrants’ fertility intentions may be decreased due to the reduction in household income following parents’ retirement, which we refer to as the "income mechanism." When the parental generation exits the labor market upon retirement, it often reduces household income, increasing the economic burden on the subsequent generation. This income reduction may increase the costs and risks of childbearing, which may have a negative impact on the fertility intentions of the subsequent generation [18–20]. In fact, the intergenerational wealth connections in developing countries are profoundly intricate [21, 22]. Notably, in China, influenced by traditional moral principles of an "intergenerational contract," parents are responsible for raising their offspring, while children are obligated to support their elderly parents [23]. The older generation not only nurtures their children to adulthood but also shoulders the tasks of securing housing, providing vehicles, and caring for their grandchildren [6]. Adult children also provide economic support and attend to the daily needs of their elderly parents [24]. It is worth noting that for the migrant population, providing childcare for retired parents entails additional household expenditures, such as housing subsidies, transportation allowances, and daily living expenses, which do not apply to local residents. Hence, there is a greater likelihood of the migrant population being influenced by income mechanisms. In developing countries like China, where the scale of public pension systems is relatively small, and where migrant populations face high fertility opportunity costs along with scarce and expensive social childcare services [20, 25], the income mechanism may take a predominant role in influencing the impact of retirement on fertility intentions. Parents’ income is a crucial source of family finances, and the income decrease resulting from retirement exacerbates the economic burden on the subsequent generation of the migrant population [26], thereby having a sustained and negative impact on fertility intentions. As the childcare support mechanism and the income mechanism work in opposite directions, how migrants’ fertility intention is affected by their parents’ retirement remains ambiguous, which depends on the relative strength of these two mechanisms.

This paper aims to address the following questions: First, how will migrants’ fertility intentions be influenced by their parents’ retirement? Second, what are the potential mechanisms driving this influence? Third, does the influence of parents’ retirement differ among migrants with different characteristics?

## Background

### Mandatory retirement system in China

The retirement age system in China is a vital component of the pension insurance framework, primarily covering urban employees. For individuals with rural hukou, the decision regarding when to exit the labor force is contingent upon their specific circumstances, and there is no legally mandated retirement age. In urban areas prior to 1978, retirement arrangements for state-owned enterprises, urban collective enterprises, government departments, and public institutions were uniform. However, after 1978, the government initiated reforms in the pension insurance systems for enterprise employees and personnel working in government and public institutions.

The retirement regulations within the enterprise employee pension insurance system differentiate based on gender and occupation, stemming from the Labor Insurance Regulations promulgated by the State Council in 1953 and the Interim Provisions on the Retirement Treatment of Workers and Staff issued by the State Council in 1958. Specifically, the standard retirement age for male employees is 60 years. However, individuals engaged in high-risk or health-hazardous occupations can retire early at the age of 55. The retirement criteria for female employees are somewhat more intricate. Women working in management and research positions, often referred to as "cadres," have a retirement age of 55, whereas the standard retirement age for ordinary female employees is 50. Similarly, those in high-risk or health-hazardous occupations can retire early at the age of 45. After 1978, pension insurance reforms mainly focused on introducing a social pooling mechanism, individual contributions, establishing a unified pension insurance system, and expanding coverage. The pension insurance system for government agencies and public institutions merged with the enterprise employee pension system in the late 1950s. In the late 1950s, the pension insurance systems for government agencies and public institutions were merged with the pension system for enterprise employees. Subsequently, they evolved into an independent pension insurance system. In addition to retaining specific provisions from the Interim Measures for the Retirement of State Organs’ Staff issued in 1955, this system primarily adhered to three documents: the Interim Measures for the Placement of Aged, Weak, Sick, and Disabled Cadres and the Interim Measures for the Retirement of Workers issued by the State Council in 1978, as well as the Interim Regulations for Civil Servants of the State enacted in 1993. As per these regulations, the standard retirement age for male civil servants is 60 years, while female civil servants retire at 55. In cases where disability is due to work-related factors, the retirement age is reduced to 55 for males and 45 for females.

Regarding employees of public institutions, the standard retirement age for males is also 60 years. Female employees, if they hold cadre positions, can retire at 55, whereas non-cadre female employees have a standard retirement age of 50. Similarly, those who become disabled due to work-related factors can retire early at 50 for males and 45 for females.

Although there are different arrangements in the pension systems for urban employees and government and public institution employees, these differences primarily pertain to the varying pension amounts. Government and public institution retirees generally receive slightly higher pensions compared to those from enterprises. However, whether in enterprises or government and public institutions, the standard retirement age for males is 60 years, and early retirement can be at either 55 or 50 years of age. The standard retirement age for females is 55 if they are cadres and 50 if they are not, with early retirement possible at 45. Except for a few exceptional cases where workers may retire early or later, most urban sector employees follow these age-based retirement procedures. After the completion of the state-owned enterprise downsizing and efficiency improvement reforms in the late 1990s, the enforcement of retirement age policies and the supervision of early retirement practices have become increasingly rigorous. Although early retirement does occur, recent household survey data reveal that the proportion of workers retiring at a younger age is minimal [27].

### Migrants in China

Furthermore, China has a large population of migrants. According to the latest *Report on the Development of China’s Migrant Population* released by the National Health Commission of China, in 2023, the scale of internal migration in China reached 247 million people, accounting for 18% of the total population. China is currently experiencing rapid urbanization, with the population continuing to concentrate on megacities and super-cities in the future. Additionally, family migration is on the rise, increasing the number of migrants accompanied by their families. In 2023, the average family size of migrant populations in their destination areas reached 2.61, showing an increase of 0.11 compared to the previous year. The age structure is becoming younger, with individuals born after 1980 representing 48.8% of the total migrant population among those in the working age category surveyed in 2023. As the primary destination for China’s migrant population, the average age of migrants in the Five Major Urban Clusters is less than 40. More than half of the migrant population consists of the new generation, and young migrants have become the predominant group in megacities.

Young migrants are increasingly willing to settle down in megacities. Nevertheless, due to restrictions imposed by China’s hukou policy, local services are often linked to hukou, making it difficult for young migrants to access essential public services, such as healthcare, housing, and employment [28]. Taking housing as an example, the *Report on the Development of China’s Migrant Population* in 2023 indicated that 67.3% of China’s migrant population rents their housing, amounting to approximately 253 million people. While young migrant groups are generally satisfied with their rented accommodations, the rent burden is heavy. Over 60% of young migrants consider rent a heavy burden, with the highest percentages among those living in privately rented commercial housing (75.54%) and long-term rental apartments (66.67%). According to the European Union’s statistical bureau standards, when a family’s housing-related expenses exceed 40% of its disposable income, it is considered one of the indicators of "housing cost overburden," which is one aspect of housing poverty. The extended rental payment periods, such as quarterly (47.18%) or semi-annually (10.92%), exacerbate the rent pressure on young migrant groups. Consequently, more than 15% of young renters face a "housing cost overburden." Therefore, migrants in China’s major cities, especially the young demographic, face significant economic challenges.

### Fertility policies in China

China’s fertility policies have undergone significant changes over the past few decades, profoundly impacting the country’s demographic landscape. The most notable of these was the One-Child Policy, implemented in 1979 to curb rapid population growth. This policy strictly limited most urban couples to having only one child, with some exceptions for rural families. In 2013, the policy was relaxed to allow couples to have two children if one parent was an only child. A more significant shift occurred in 2016 when China officially ended the One-Child Policy, allowing all couples to have two children. This change was motivated by concerns about an aging population and declining workforce. Most recently, in May 2021, China further relaxed its policy to allow couples to have up to three children, responding to the country’s ultra-low fertility rates and rapidly aging population.

It’s important to note that while these national policies apply to all Chinese citizens, their implementation and impact can differ for internal migrants. Migrants often face additional challenges in accessing public services, including healthcare and education, which can influence their fertility decisions. Some local governments have implemented policies to encourage childbirth among migrants, such as extending maternity leave or providing childcare subsidies, but these vary by region and are often not as comprehensive as those available to local residents. Despite policy changes encouraging higher fertility, socioeconomic factors such as high living costs, especially in urban areas, continue to influence fertility decisions significantly, particularly among the migrant population who often face greater economic pressures.

## Data and measurement

### Data

This study uses data from the China Migrants Dynamic Survey (CMDS). CMDS is a large-scale survey initiated by the National Health Commission of China. It focuses on gathering data from a nationally representative sample of migrant populations across all 31 provinces, autonomous regions, municipalities, and areas with concentrated migrant populations, such as Xinjiang. The survey targets individuals aged 15 and above who have resided in their current location for one month or longer and do not possess local hukou. CMDS data undergo rigorous quality control procedures and employ a stratified, multi-stage, and proportional-to-size probability sampling method. The survey is designed to address various social issues relevant to the migrants. The CMDS is publicly available to authorized researchers who have been given permission by the Migrant Population Service Center, and written informed consents were obtained from all participants. The analysis of public access data was exempted by the local IRB; as this involved analyzing de-identified existing data, ethical approval was not required.

The dataset boasts substantial yearly sample sizes, with 200,937 individual records available in 2014 and 152,000 in 2018. This robust sample size is crucial for the regression-discontinuity design used in this study, which relies on a narrow age range. The survey covers extensive information, including basic demographic details, migration patterns, reasons for migration, employment and social security, household income and expenditures, essential public health services, marital status, fertility, and family planning practices. It also collects detailed information about the respondent’s family members, including parents and spouses. These comprehensive data elements are highly pertinent to our study’s objectives. CMDS data serve as a solid foundation for our research, facilitating the RDD and offering a rich dataset that accurately represents the diverse migrant population in China.

### Sample

**Fig 1:**
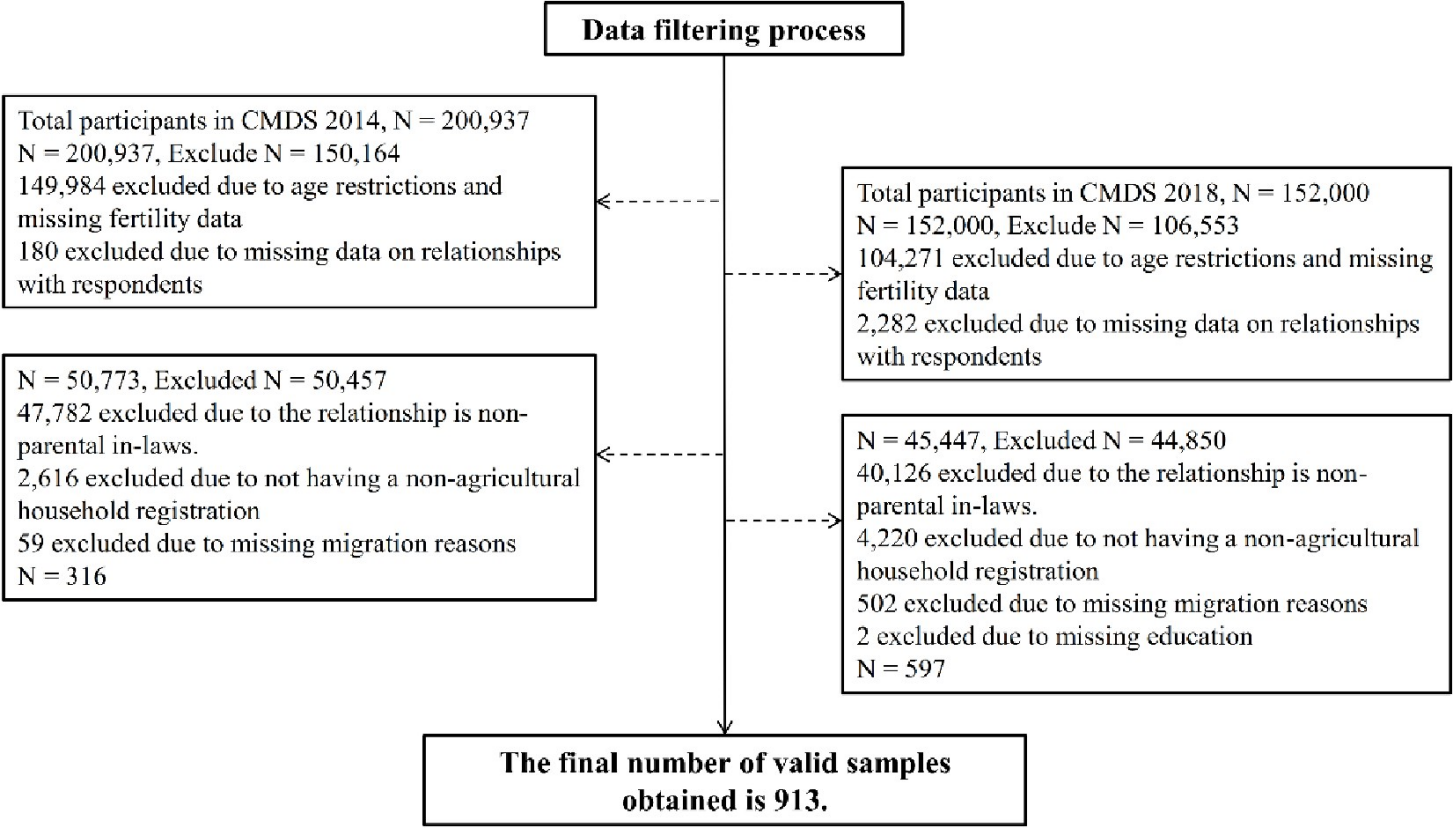
Sample selection process.

Due to the limited availability of information regarding migration and fertility intentions among the migrant population, this study combines data from the CMDS for 2014 and 2018. The CMDS is an individual-level survey that collects detailed information about respondents’ family members, including parents and spouses. Given that the minimum marriage age is 20 years for women, and fertility in China of married women in their 40s is incredibly low, the sample used in this research is restricted to married women aged 20 to 39 and their spouses. The parental generation local co-resident with their adult children must meet two conditions to account for the influence of retirement age policies: (1) Urban hukou: This condition refers to the parental generation with an urban hukou instead of a rural hukou. In China, the urban employee pension insurance system consists of two categories: one for urban enterprise employees and another for public institution employees. This condition primarily applies to urban areas in China. Rural residents can decide to exit the labor market according to their own circumstances and are not subject to a statutory retirement age. Therefore, they are not included in the sample. The research object of this article is the migrant population from the city to the city, not the rural-to-city. (2) Permanent Exit from the Labor Market: Their migration reasons are considered to determine whether parents have permanently left the labor market. If respondents state that their migration reason is "work" or "business," they have not exited the labor market. Conversely, mentioning reasons such as "accompanying family" or "retirement in another location" signifies that they have retired. Additionally, the study restricts the parental generation sample around the retirement age threshold. Parents aged 40 to 60 are considered for the female sample, while for the male sample, the age range is from 50 to 70. After thorough data cleaning, a total of 913 valid samples were obtained. This sample selection process is illustrated in Fig 1.

### Measurements

#### Dependent variable

The dependent variable is the fertility intention of the migrant population. Since fertility intentions were only asked of married women, we use the wife’s intention as the measure of offspring fertility intentions, based on the assumption that couples typically have consistent fertility intentions. It is measured based on the question: "Do you plan to have children in the next two years?" There are four response options: 1. Yes 2. No 3. Undecided 4. Pregnant. Respondents who answer "Yes" or "Pregnant" indicate a willingness to have children; the indicator variable is set to 1. If the answer is "No," it indicates no willingness to have children, and the indicator variable is set to 0. Samples with an answer of "Undecided" are deleted.

#### Independent variable

The independent variable in this study is the parental retirement status. Assuming the retirement status of parents is the independent variable, with the forcing variable affecting whether they receive treatment being the age of the parental sample. When the age of the parents exceeds or equals the legal retirement age, they are affected by retirement age policies (*r*_*i*_ = 1); conversely, they are not affected (*r*_*i*_ = 0). Parental retirement status is determined following Feng (2020). Specifically, we consider which parent or parent-in-law is closer to the legal retirement age. For instance, if a respondent’s mother (legal retirement age 50) is 51 and the father (legal retirement age 60) is 56, we use the mother’s retirement status to represent the parental generation’s retirement status.

#### Control variables

To control for the influence of parental retirement age, the difference between the actual age of the parental generation and the statutory retirement age is calculated as *T*_*i*_. The time distance from the statutory retirement age is based on the age of the parental generation covered by the urban employee pension insurance system. If both parents are covered, the one closer to the statutory retirement age is used. The offspring’s age also impacts fertility intentions, so it is controlled in the equation. Other control variables include a series of individual and family characteristics that may affect the offspring’s fertility intentions. Individual characteristics include age, education level, marital status and migrant range of the offspring. To avoid potential multicollinearity issues, given that married couples in migrant populations often have highly similar characteristics, we only included the individual characteristics of the wife. As for family characteristics, number of existing children, sex of existing children, average monthly household income are included. Moreover, to account for potential effects of the 2016 fertility policy change, a dummy variable for the survey year (2018 vs. 2014) was included in all regression models.

### Descriptive statistics

Table 1 presents the descriptive statistics for the main variables. Panel A displays the family characteristics. Of the households, 70.65% include a father or father-in-law, while 87.19% have a mother or mother-in-law. Additionally, 57.83% of households have both parents living together. Regarding children, 57.28% of households have at least one boy, 46.77% have at least one girl, and 10.51% have both. The majority (73.93%) have one child, while 19.61% have two or more children, and 6.46% are childless. The average number of existing children is 1.14. Economically, the mean monthly household income is 3062.98 yuan, with average monthly expenses of 1354.65 yuan.

**Table 1:** Descriptive statistics of key variables.

		N	Mean/Proportion	SD
Panel A: Family characteristics				
Families with father or father-in-law		645	70.65%	
Families with mother or mother-in-law		796	87.19%	
Families with both parents		528	57.83%	
Families with boy		523	57.28%	
Families with girl		427	46.77%	
Families with both boy and girl		96	10.51%	
Families with 0 child		59	6.46%	
Families with 1 child		675	73.93%	
Families with 2+ children		179	19.61%	
Average monthly household income		913	3062.98	5133.81
Average monthly household expenses		913	1354.65	966.63
Number of existing children		913	1.14	0.51
Panel B: Parental generation				
Age	Age of male	645	59.43%	6.68
	Age of female	796	57.37%	6.24
Migrant reason (male)	Work/Business	176	27.29%	
	Accompanying family members	469	72.71%	
Migrant reason (female)	Work/Business	129	16.21%	
	Accompanying family members	667	83.79%	
Panel C: Subsequent generation				
Age		913	30.90	4.25
Education	Elementary school and below	15	1.64%	
	Junior high school	182	19.93%	
	Senior high school	179	19.61%	
	College and above	537	58.82%	
Marital status	First-marriage	889	97.37%	
	Remarriage	24	2.63%	
Migrant range	Interprovincial	521	57.06%	
	Intermunicipal	260	28.48%	
	Intercounty	132	14.46%	
Fertility intention		913	0.24	0.43

Notes

a. Based on the CMDS 2014 and 2018 surveys, Panel B compiles statistics on a sample of parents or parents-in-law covered by the urban employee pension insurance system. The sample includes data from ten years before and after the statutory retirement age.

b. Panel C compiles statistics on the offspring sample matched with the parental sample. Average monthly household income and average monthly household expenditure refer to the total income and expenditure of the household in their current place of residence each month. The willingness to have children is determined by the question, "Do you have plans to have children in the next two years?".

Panel B displays the basic characteristics of the parental generation sample. The average age of the male sample is 59.43 years, situated around the statutory retirement age threshold. The average age of the female sample is 57.37 years. Regarding migration reasons, there’s a notable gender difference. For male parents, 27.29% migrated for work or business purposes, while 72.71% moved to accompany family members. In contrast, female parents show a stronger tendency towards family-oriented migration, with only 16.21% moving for work or business, and a substantial 83.79% migrating to accompany family members.

Panel C presents the statistical characteristics of the offspring sample. The average age of the offspring sample is 30.90 years, within the childbearing age range. However, the average fertility intention is only 0.24, indicating a generally low overall fertility intention in the sample. Regarding the offspring’s educational level, the majority have a college degree or higher (58.82%), while only a few have primary school education or below (1.64%). Regarding marital status, the vast majority are in their first marriage (97.37%), with only a small percentage in remarriage (2.63%). In terms of migration range, most of the respondents have migrated interprovincially (57.06%), followed by intercity (28.48%) and intercounty (14.46%) migrates.

## Empirical strategy

In this study, we exploit a regression discontinuity design (RDD) to estimate the local average treatment effect of parental retirement on the fertility intentions of the migrant population offspring. This method is based on the intuition that the distribution of retirement status around the cutoff point appears to be random. Around the retirement age cutoffs, retired and unretired parents are very similar. Therefore, comparing the fertility intentions of offspring with retired parents and unretired parents within sufficient proximity to the cutoff can obtain an unbiased local causal estimation of the relationship between migrants’ fertility intention and their parents’ retirement status. The retirement age system is reflected by whether the parent has reached the statutory retirement age. However, not everyone stops working at the prescribed age. There may be cases of early retirement due to health conditions or re-employment after retirement procedures. Therefore, our study utilizes a fuzzy regression discontinuity approach.

Table 2 conducts local smoothness test of covariates. Each covariate is treated as an outcome variable, and a regression discontinuity approach is employed for examination. The results reveal that, apart from the offspring age, all other variables exhibit no treatment effects in the vicinity of the cutoff, showing no discontinuities or jumps.

**Table 2:** Local smoothness test of covariates.

Variables	Education	Marital status	Migrant range	Offspring age	Household income
RD_Estimate	0.032	-0.001	-0.015	2.124*	2.041
	(0.228)	(0.004)	(0.191)	(1.157)	(1.548)
Variables	Number of children	Ever have boy	Ever have girl	Ever have boy and girl	
RD_Estimate	-0.020	-0.190	0.243	0.115	
	(0.145)	(0.174)	(0.183)	(0.112)	

Notes

a. This table treats each covariate as a placebo outcome variable and demonstrates the absence of treatment effects for other variables near the cutoff.

b. Standard errors in parentheses *** p<0.01, ** p<0.05, * p<0.1

The fuzzy regression discontinuity (FRD) model can be estimated using a two-stage least squares (2SLS) approach, with an exogenous mandatory retirement age system as an instrument variable, to identify the impact of retirement on fertility intentions. As demonstrated in previous studies, FRD can be achieved through 2SLS, which is equivalent to instrumental variable (IV) estimation [29, 30]. 2SLS is an effective method for addressing endogeneity issues [31, 32]. Utilizing ordinary least squares (OLS) estimation with the raw variable "retirement" would result in biased estimates [33]. However, since retirement is highly correlated with age, and age is a clearly defined and highly exogenous variable, using age-related instrument variables allows for a more precise estimation of the retirement effect. In this study, a two-stage least squares method is employed, with the first stage utilizing "whether exceeding the statutory retirement age" as the instrument variable for retirement and the second stage integrating the instrument variable into the model to address endogeneity concerns. The following FRD design of the effect of retirement on fertility intentions is conducted through two equations of 2SLS:

Effects of Stage 1 of retirement age on retirement behavior:

$$r_{i}=\beta_{0}+\beta_{1}R_{i}+\beta_{2}T_{i}+\beta_{3}R_{i}T_{i}+X_{i}\gamma +\varepsilon_{i}$$ (1)

Effects of Stage-2 of retirement behavior on fertility intentions of their offspring:

$${\mathrm{birth}}_{i}=\pi_{0}+\pi_{1}{\hat{r}}_{i}+\pi_{2}T_{i}+\pi_{3}T_{i}R_{i}+X_{i}\rho +\zeta_{i}$$ (2)

In the first stage, the variable *r*_*i*_ represents "whether actually retired" (hereafter referred to as *actual retirement*). *R*_*i*_ represents whether the parental generation surpasses the statutory retirement age, taking the value 1 if surpassed and 0 otherwise. *T*_*i*_ represents the age difference at the cutoff (sample age—statutory retirement age), used to control for the influence of the parental generation’s age. The research employs a linear model specification, incorporating interaction terms, allowing for differing age effects on both sides of the cutoff. Additionally, a series of control variables were included to account for individual characteristics that could potentially influence the fertility intentions of the offspring. These control variables encompass factors such as the children’s educational attainment, marital status, migration range, age of the children, and per capita monthly household income.

**Fig 2:**
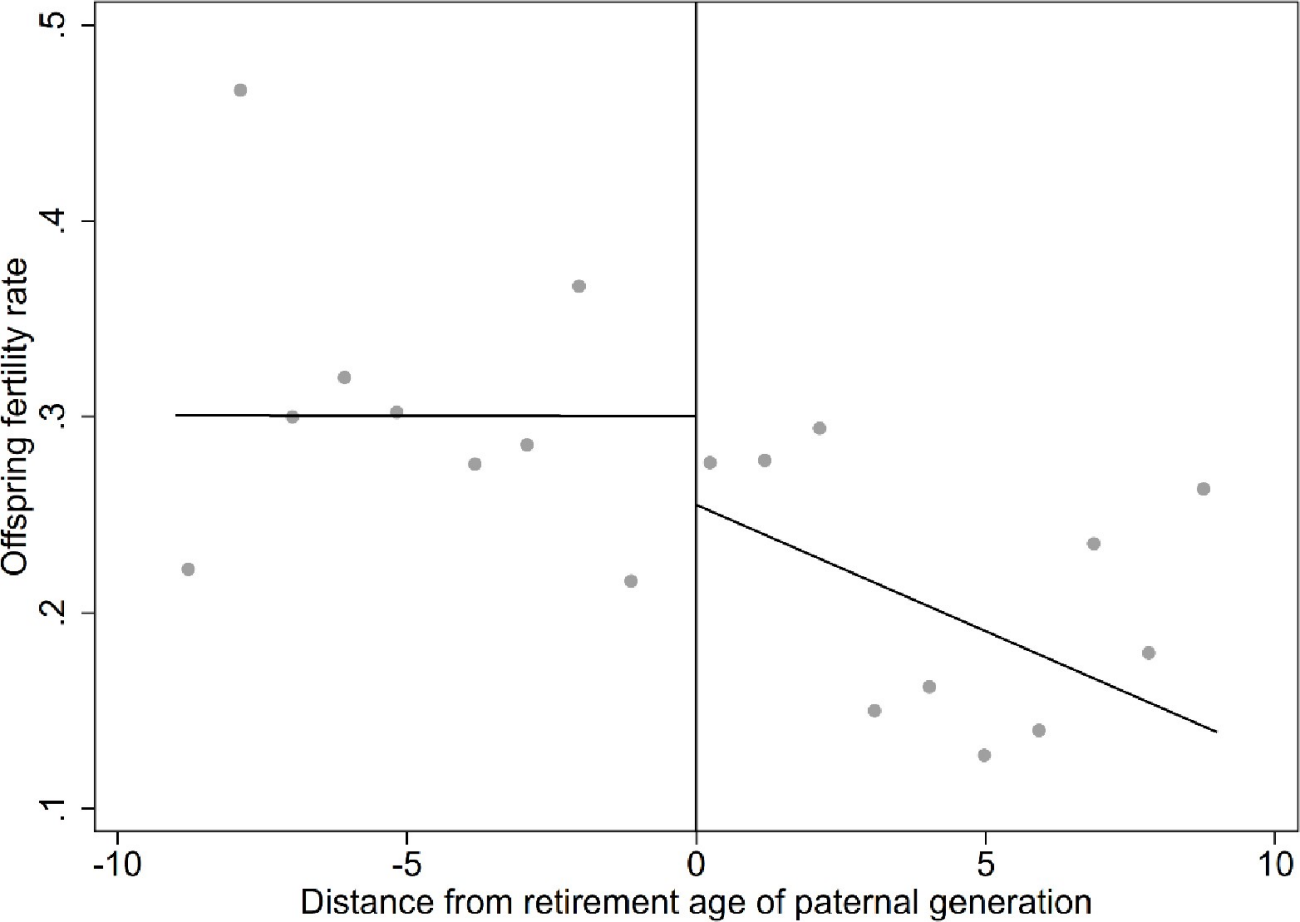
Relationship between distance from retirement age of parental generation and fertility rate of offspring. Notes: a. The sample consists of parents covered by the urban employee pension insurance system and within ten years before or after their retirement age, along with married women aged 20–39 and their spouses among the offspring. b. The distance from the statutory retirement age for parents is determined based on whichever side is covered by the system. If both are covered, the determination is based on the one closer to the statutory retirement age.

Fig 2 concisely depicts the changing trend in offspring fertility intentions before and after the cutoff. It is evident that, overall, the fertility intentions of the offspring gradually decrease with parent age. However, when the parental generation has not yet reached the statutory retirement age, the fertility intentions of the offspring remain relatively stable. A notable jump occurs at the statutory retirement age cutoff. As the parental generation surpasses the statutory retirement age, the fertility intentions of the offspring decrease and exhibit a declining trend. This finding suggests that the impact of the parental generation reaching retirement age on the fertility intentions of the offspring is a plausible phenomenon.

## Results

### Baseline effect of retirement age policy

Table 3 checks the correlations between parents surpassing the retirement age and their actual retirement status. It is evident that whether the parental generation surpasses the retirement age significantly affects their actual retirement. Exceeding the statutory retirement age increases the probability of actual retirement by 10.2%, which is statistically significant at the 1% level.

**Table 3:** Impact of the retirement system on actual retirement.

Variables	Actual retire	Actual retire	Actual retire	Actual retire	Actual retire	Actual retire
R	0.102***	0.102***	0.101***	0.101***	0.101***	0.102***
	(0.034)	(0.034)	(0.034)	(0.034)	(0.035)	(0.035)
T×R	0.030***	0.030***	0.030***	0.030***	0.031***	0.031***
	(0.005)	(0.005)	(0.005)	(0.005)	(0.006)	(0.006)
T^2^	-0.002***	-0.002***	-0.002***	-0.002***	-0.002***	-0.002***
	(0.000)	(0.000)	(0.000)	(0.000)	(0.000)	(0.000)
Education	0.076***	0.075***	0.073***	0.073***	0.075***	0.075***
	(0.014)	(0.014)	(0.015)	(0.015)	(0.015)	(0.015)
Marital status		0.052	0.055	0.055	0.050	0.047
		(0.076)	(0.076)	(0.076)	(0.076)	(0.076)
Migration range			-0.016	-0.016	-0.016	-0.013
			(0.017)	(0.017)	(0.017)	(0.017)
Household income				0.000	0.000	0.000
				(0.002)	(0.002)	(0.002)
Offspring age					-0.001	-0.001
					(0.004)	(0.004)
Number of children					0.027	0.042
					(0.025)	(0.043)
Ever have boy						0.023
						(0.072)
Ever have girl						0.002
						(0.073)
Ever have boy and girl						-0.066
						(0.071)
Year of 2018	-0.039	-0.038	-0.039	-0.039	-0.045*	-0.038
	(0.025)	(0.025)	(0.025)	(0.025)	(0.026)	(0.028)
Constant	0.505***	0.457***	0.488***	0.488***	0.496***	0.474***
	(0.056)	(0.089)	(0.094)	(0.095)	(0.142)	(0.146)
Observations	913	913	913	913	913	913

Notes

a. This table represents the estimation of Eq (1).

b. Each column represents an individual regression, systematically introducing control variables one by one to examine the impact of the retirement system on actual retirement.

c. Standard errors in parentheses *** p<0.01, ** p<0.05, * p<0.1

### Baseline effect of actual retirement

Table 4 examines the impact of actual retirement on the fertility intentions of the offspring of the migrant population. It is revealed that, for the migrant population, whether the parental generation exceeds the statutory retirement age has a statistically significant adverse effect on the fertility intentions of their offspring. As more control variables are introduced, the regression results remain robust. When the parental generation exceeds the retirement age, it reduces the fertility intentions of their offspring by approximately 34.4%. Additionally, there is a significant negative relationship between the age of the offspring and their fertility intentions, consistent with the findings in Fig 2.

**Table 4:** Effect of parental retirement on offspring’s fertility intentions.

Variables	Birth	Birth	Birth	Birth	Birth	Birth
r	-0.565***	-0.575***	-0.577***	-0.583***	-0.304**	-0.344**
	(0.125)	(0.125)	(0.127)	(0.127)	(0.155)	(0.152)
T^2^	-0.000	-0.000	-0.000	-0.000	-0.000	-0.000
	(0.000)	(0.000)	(0.000)	(0.000)	(0.000)	(0.000)
Education	0.063***	0.071***	0.071***	0.063***	0.018	0.020
	(0.022)	(0.022)	(0.022)	(0.022)	(0.021)	(0.021)
Marital status		-0.350***	-0.350***	-0.355***	-0.278***	-0.257***
		(0.097)	(0.097)	(0.097)	(0.085)	(0.084)
Migration range			-0.004	0.005	0.003	-0.006
			(0.022)	(0.022)	(0.019)	(0.019)
Household income				0.010***	0.010***	0.009***
				(0.003)	(0.003)	(0.003)
Offspring age					-0.010**	-0.008*
					(0.005)	(0.005)
Number of children					-0.264***	-0.111**
					(0.028)	(0.047)
Ever have boy						-0.473***
						(0.079)
Ever have girl						-0.356***
						(0.080)
Ever have boy and girl						0.339***
						(0.078)
Year of 2018	-0.092***	-0.096***	-0.096***	-0.104***	-0.037	-0.099***
	(0.033)	(0.033)	(0.033)	(0.033)	(0.029)	(0.031)
Constant	0.561***	0.886***	0.894***	0.892***	1.297***	1.531***
	(0.096)	(0.128)	(0.139)	(0.139)	(0.141)	(0.145)
Observations	913	913	913	913	913	913

Notes

a. This table represents the estimation of Eq (2).

b. Each column represents a separate regression, progressively adding control variables to examine the impact of actual retirement on fertility.

c. Standard errors in parentheses *** p<0.01, ** p<0.05, * p<0.1

### Robustness checks

To test whether the bandwidth affects parameter estimates, we narrowed the window on both sides of the cutoff, adjusting the bandwidth to 8 years, 9 years, and 10 years before and after the cutoff. The coefficients remained statistically significant. Moreover, as the bandwidth decreased, the absolute value of the coefficients increased. This finding implies that a smaller bandwidth corresponds to a larger negative impact of parental retirement beyond the retirement age on the fertility intentions of offspring among the migrant population. The results are presented in Table 5.

**Table 5:** Effect of parental exceeding retirement age on offspring’s fertility intentions.

Variables	Bandwidth 8 years	Bandwidth 9 years	Bandwidth10 years
r	-0.346*	-0.306*	-0.308*
	(0.204)	(0.181)	(0.176)
T^2^	-0.001	-0.001*	-0.001*
	(0.001)	(0.001)	(0.001)
Covariates	Controlled	Controlled	Controlled
Constant	1.692***	1.527***	1.528***
	(0.202)	(0.177)	(0.170)
Observations	730	785	802

Notes

a. Varying different bandwidths, regressing with Eq (2).

b. Standard errors in parentheses *** p<0.01, ** p<0.05, * p<0.1

**Fig 3:**
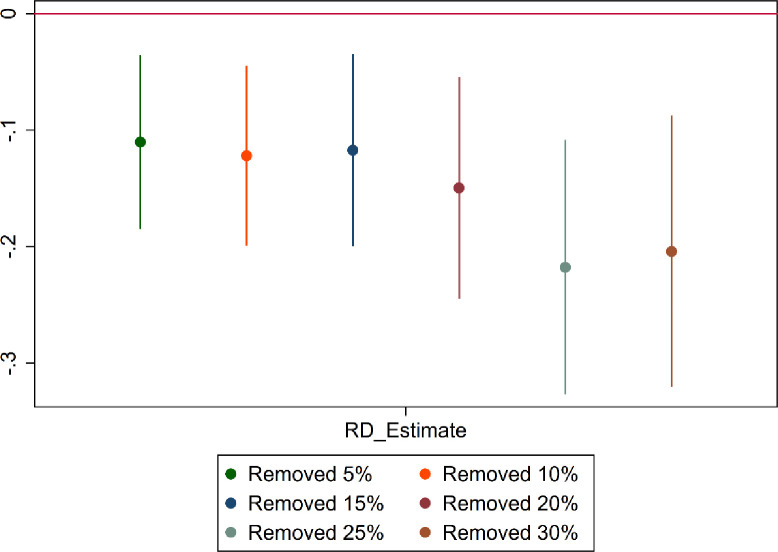
Sensitivity test for sample selection. Notes: This figure depicts six sets of robustness tests, each involving the removal of samples within 5%, 10%, 15%, 20%, 25%, and 30% around the breakpoint. The graph presents regression coefficients and 95% confidence intervals.

Considering that individuals closer to the cutoff age may have a greater incentive to manipulate the results artificially, we perform donut regression discontinuity estimation to examine if our results are driven by observations close to the cutoff ages. Specifically, we removed the samples closest to the cutoff point. If the regression coefficients remain significant, it suggests that the cutoff effect persists even in the presence of potential manipulation. The test results are shown in Fig 3, which presents the regression coefficients and 95% confidence intervals. We conducted six robustness tests by successively removing 5%, 10%, 15%, 20%, 25%, and 30% of the samples near the cutoff, and in all cases, the regression results remained significant.

## Potential mechanisms

The baseline results indicate that, for the migrant population, parents’ retirement somewhat hurts the fertility intentions of their offspring. In this section, we attempt to analyze the mechanisms through which parents’ retirement affects the fertility intentions of their offspring, considering both the "income mechanism" and "childcare support mechanism."

### Income mechanism

If their parents’ retirement mainly influences migrants’ fertility intention through income mechanism, the negative impact of parents’ retirement may be less prominent in wealthier families. We use two indicators, per capita monthly household income and per capita monthly household expenditure, to test for the income mechanism. These are crucial metrics for assessing individual and family economic circumstances. We obtained these variables by dividing the original survey data on the total monthly household income and total monthly household expenditure by the number of individuals in the household.

We divided the sample into low-income, middle-income, and high-income groups based on per capita monthly household income and repeated analysis in Table 4 for each group. The results are shown in Table 6. The coefficient of parents’ retirement is significantly negative for the low-income group. This coefficient is negative but insignificant for the middle-income group and is positive but insignificant for the high-income group. This finding aligns with our expectations, that is, the negative impact of parents’ retirement on migrants’ fertility intention is more prominent in families with lower income. After retirement, migrant parents usually receive pensions lower than their pre-retirement wages, reducing household income. This effect is more pronounced for low-income families, where the parental generation’s wage income may be a significant part of the household’s economic resources, making them more vulnerable to negative impacts. While the impact on middle-income families is negative but not statistically significant, and the impact on high-income families is also not statistically significant, the coefficient becomes positive. This finding may be explained by the positive incentive effect of intergenerational childcare support associated with parents’ retirement.

**Table 6:** Heterogeneity test of the impact of parental retirement on subsequent generational fertility intentions with varied household per capita monthly income.

Variables	Low income	Middle income	High income
r	-0.449**	-0.068	0.613
	(0.177)	(0.305)	(1.533)
T^2^	-0.000	-0.000	-0.001
	(0.000)	(0.001)	(0.010)
Covariates	Controlled	Controlled	Controlled
Constant	1.449***	2.051***	1.592
	(0.164)	(0.499)	(1.816)
Observations	763	118	32

Notes

a. The sample selection is the same as in Table 1.

b. It is divided into three groups based on household per capita monthly income, and regression is performed using Eq (2).

c. Standard errors in parentheses *** p<0.01, ** p<0.05, * p<0.1

Based on the *monthly total family income* calculated from the questionnaire, the *per capita monthly family income* encompasses wage, business, property, and transfer income. However, this measure may not be sufficiently sensitive to reflect the economic status of households dynamically. In this study, we also constructed an indicator of *average monthly family expenditure* to examine the role of wealth transfer mechanisms in retirement and fertility intentions. Expenditure in this context includes all relevant cost outlays incurred by family members in their current residence. As shown in Table 7, the coefficient for families in the low expenditure group is significantly negative, while for families in the high expenditure group, it is significantly positive, with no significant coefficient observed for families in the medium expenditure group. The negative impacts of parents’ retirement on migrants’ fertility intentions are the most prominent for families with the lowest expenditure level. However, for families with better economic conditions, parents’ retirement positively influences the fertility intentions of the migrants. These results are qualitatively in line with the results using the per capita monthly household income, further supporting the argument of the income mechanism.

**Table 7:** Heterogeneity test of the impact of parental retirement on subsequent generational fertility intentions with varied household per capita monthly expenditure.

Variables	Low expenditure	Middle expenditure	High expenditure
r	-0.421**	-0.626	1.341**
	(0.176)	(0.510)	(0.613)
T^2^	0.000	-0.001**	0.000
	(0.000)	(0.001)	(0.001)
Covariates	Controlled	Controlled	Controlled
Constant	1.478***	1.849***	0.943
	(0.171)	(0.541)	(0.700)
Observations	637	199	77

Notes

a. The sample selection is the same as in Table 1.

b. It is divided into three groups based on household per capita monthly expenditure, and regression is performed using Eq (2).

c. Standard errors in parentheses *** p<0.01, ** p<0.05, * p<0.1

### Childcare support mechanism

Most literature suggests that retirement allows grandparents to allocate more time to help care for their grandchildren, and this informal caregiving helps alleviate the fertility constraints on young women. The indicator of *Reason for Parents’ Migration—Family Relocation—Childcar*e is employed to examine the role of this mechanism. We divide the migrants into the *Childcare Group* and *Non-Childcare Group* based on their parents’ reason for migration and repeat the same analysis in Table 4 for each group respectively. The results are presented in Table 8. It is revealed that for individuals who migrated for reasons other than childcare, retirement has a more negative impact on their fertility intentions compared to those who migrated for the purpose of childcare.

**Table 8:** Heterogeneity test for migration reasons.

Variables	Childcare Group	Non-Childcare Group
R	-0.003	-0.061
	(0.056)	(0.048)
T×R	-0.004	-0.012
	(0.010)	(0.007)
T^2^	-0.001	0.001
	(0.001)	(0.000)
Covariates	Controlled	Controlled
Constant	1.129***	1.430***
	(0.221)	(0.209)
Observations	339	574

Notes

a. The sample selection is the same as in Table 1.

b. It is divided into two groups based on migration reasons, and regression is performed using Eq (2).

c. Standard errors in parentheses *** p<0.01, ** p<0.05, * p<0.1

The above analysis demonstrates that intergenerational childcare support may incentivize migrants’ fertility intention. However, in this study, we find that the negative income mechanism generated by parents’ retirement is stronger than the positive effect of intergenerational childcare support, ultimately decreasing migrants’ fertility intentions.

## Discussion

Existing literature often discusses the intergenerational impact on fertility intentions and the fertility intentions of migrant populations separately. This study combines the two aspects to explore the influence of parents’ retirement on the fertility intentions of migrant offspring, providing a supplementary perspective to the existing literature. The findings of this study suggest that parents’ retirement has a negative impact on the fertility intentions of migrant offspring, a finding that has not been documented in the existing literature [3, 34].

One possible explanation for this negative impact is that parents’ retirement not only increases intergenerational childcare support but also reduces economic support due to the interruption of wage income. For migrants, the decreased fertility intention in response to the income reduction outweighs the increased fertility intention associated with increased intergenerational childcare support, leading to an average decrease in fertility intentions. Our empirical analysis finds that the fertility intentions of migrants living in less affluent economic conditions are more adversely affected by their parents’ retirement, supporting our argument of the "income mechanism" channel. Moreover, the analysis of intergenerational child support suggests that for households currently without children or with only one child, retirement does not have a significant negative impact on the fertility intentions of the younger generation; for households with two or more children, the negative influence of retirement on the fertility intentions of the younger generation becomes significant. This finding could be attributed to the increased challenges in parental childcare provision, lending support for the "time mechanism" mechanism. This finding is consistent with the extant research [14, 16, 34]. In summary, we find that the impact of parents’ retirement on the fertility intentions of migrant populations depends on the relative strength of the childcare support mechanism and time mechanism, to some extent resonating with the findings from previous research [18]. Our study suggests that migrants’ fertility intentions are, on average, negatively impacted by their parents’ retirement because the income mechanism outweighs the childcare support mechanism.

The theoretical implications of this study encompass several vital points. First, this research contributes to the literature on fertility intentions among the migrant population. Existing studies on the intergenerational impact on fertility intentions have overlooked the heterogeneity within the target population [6, 35–37]. However, for local residents and the migrant population, the influence of parents’ retirement on fertility intentions differs significantly. Migrant populations, due to the separation between their current residence and registered residence, incur higher costs associated with childbearing and often lose certain benefits tied to their registered residence. Additionally, the migrant population may face the financial burden of housing, transportation, and daily expenses for their parents who come to help with childcare, costs not borne by local residents. Consequently, this paper further refines the study population, providing a more detailed examination of the substantial migrant population and their fertility intentions. Second, this research contributes to the literature on the income mechanism. Previous studies exploring the impact of parents’ retirement on the fertility intentions of the next generation have primarily emphasized the role of intergenerational childcare support [34, 38]. In contrast, our study adds to this literature by emphasizing the role of the income mechanism. We argue that the influence of parents’ retirement on the fertility intentions of the next generation is contingent on the relative strength of income and intergenerational childcare support mechanisms. Moreover, for the migrant population, the income mechanism’s negative impact outweighs the benefits of childcare support mechanisms. This conclusion aligns with the consensus reached by some scholars in the literature that examines the relationship between grandparents’ labor force participation and the fertility intentions of their offspring. This literature suggests that the non-linear relationship between elderly labor force participation and fertility intentions can be attributed to the dual role of parents in the labor market and household production [20]. While participation in the labor force may provide less childcare, their offspring are more likely to have children, as parents’ labor force participation leads to increased family income, offsetting the reduced costs of childcare [18]. From the perspective of parents’ retirement, this study underscores the vital role of the income mechanism.

Furthermore, this study bears policy implications and practical insights. First, our research indicates that parents’ retirement can negatively influence the fertility intentions of migrant offspring. This underscores the need for policies that support migrant families in their unique circumstances, which may include addressing issues related to housing, healthcare, and education access. Second, this paper confirms the positive impact of informal support from retired parents on the fertility intentions of the offspring. However, this effect is constrained by the age and health of the parents, and its positive influence is somewhat limited. It’s important to note that while grandparental care can be beneficial, it may also have potentially exploitative aspects. The reliance on retired parents for childcare could place undue burden on the older generation, potentially compromising their well-being and autonomy [39]. Thus, policymakers should consider ways to support intergenerational caregiving that benefit both the older and younger generations. This could include programs that promote active ageing through grandparental care, while also providing respite care and support services to prevent burnout and exploitation. To address the diverse needs identified in our study, we propose a multi-faceted approach to family support policies. This could include: developing flexible childcare options that cater to the varied needs of different families, including migrants; providing training and resources for childcare professionals to improve the quality and availability of formal childcare services; creating community programs that foster intergenerational connections and shared caregiving responsibilities. Third, when implementing policies related to delayed retirement age, it is imperative to consider their potential impact on fertility issues and ensure the welfare of the delayed retirement population. Rather than focusing solely on fertility rates, policymakers should aim to create an environment where individuals and families feel supported in their choices, whether that involves having children, providing care for family members, or pursuing other life goals. Ultimately, the goal should be to develop a comprehensive support system that recognizes the diverse needs of different populations, promotes intergenerational solidarity, and enhances overall quality of life for all members of society.

This study has two limitations. Firstly, due to data constraints, it exclusively considers urban parental populations co-residing with their offspring. Consequently, the research does not encompass the effects of parents’ retirement on those with rural hukou or those engaged in lower-skilled labor, potentially leading to the underestimation of the income mechanism. Secondly, we cannot provide a direct test for the two mechanisms as our data set does not contain detailed information on parenting hours and intergenerational income transfer. In future research, if data allows, more extensive and detailed analysis is needed for testing these mechanisms.

## Conclusion

Population aging and continuously declining birth rates are common challenges faced by countries worldwide today. Utilizing the mandatory retirement age system as an exogenous shock within a fuzzy regression discontinuity experimental design, this study examines the impact of parents’ retirement on the fertility intentions of their migrant offspring and explores the underlying mechanisms. The findings of the study are as follows.

For the migrant population, parents’ retirement has a negative impact on the fertility intentions of their offspring. Specifically, parents’ retirement reduces the fertility intentions of the younger generation by 34.4%. We further investigate the underlying mechanisms of this negative impact. Heterogeneous analysis shows that the impacts of parents’ retirement are significantly negative for the low-income/expenditure group and positive for the high-income/expenditure group are positive. This finding suggests that the negative impact of parents’ retirement on the fertility intentions of the younger generation is more pronounced among families in poor economic conditions. These findings suggest the mediating role of the "income mechanism" in the relationship between migrants’ fertility intentions and their parents’ retirement status.

Further, we find that for parents who migrate to take care of their grandchildren, the negative impact of retirement on fertility intentions is not significant. However, for parents not providing intergenerational childcare support, the negative impact of parents’ retirement is more pronounced. These results provide evidence for the role of intergenerational childcare support.

In conclusion, this study posits that the impact of parents’ retirement on the fertility decisions of the migrant population depends on the relative strengths of income and childcare support mechanisms. In this study, among migrant families, parents’ retirement negatively influences the fertility intentions of the younger generation on average, which is attributed to the stronger effects coming from the income mechanism. This study has theoretical and practical implications and offers recommendations for family support policies to improve migrants’ welfare.
